# Diversity and Bioactivity of Endophytes From *Angelica sinensis* in China

**DOI:** 10.3389/fmicb.2020.01489

**Published:** 2020-08-18

**Authors:** Xin-Guo Zhang, Si-Jia Guo, Wen-Na Wang, Guo-Xing Wei, Guo-Yan Ma, Xiao-Di Ma

**Affiliations:** ^1^School of Life Sciences and Engineering, Lanzhou University of Technology, Lanzhou, China; ^2^Key Laboratory of Screening and Processing in New Tibetan Medicine of Gansu Province, Gansu, China

**Keywords:** *Angelica sinensis*, endophytes, diversity, secondary metabolite, antimicrobial activity, antioxidant activity

## Abstract

Plant seeds are not merely reproductive organs, they are also carriers of microorganism, particularly, inherent and non-invasive characteristic endophytes in host plant. Therefore, in this study, the endophytic diversity of *Angelica* seeds was studied and compared with endophytes isolated from healthy leaves, stems, roots, and seeds of *A*. *sinensis* using 20 different media. The metabolites of endophytic strains were evaluated with six different methods for their antioxidant activity and the paper disc diffusion method for antimicrobial activities. As a result, 226 endophytes were isolated. Compared with the biodiversity and abundance of uncultured fungi from *Angelica* seed, the result showed that the most frequent endophytic fungi were *Alternaria* sp. as seen in artificial media; moreover, compared with artificial media, the pathogenic fungi, including *Fusarium* sp. and *Pseudallescheria* sp., were not found from the *Angelica* seed, the results suggested it may not be inherent endophytes in plants. In addition, bacteria from seven phyla were identified by high-throughput sequencing, while five phyla of endophytic bacteria were not isolated on artificial media including *Proteobacteria*, *Actinobacteria*, *Bacteroidetes*, *Microgenomates*, and *Saccharibacteria*. Furthermore, the sample JH-4 mycelium displayed the best antioxidant activity, and the active constituent may be a flavonoid as determined by total phenol and flavonoid content. Moreover, YH-12-1 mycelium had strong inhibitory activity against the five tested strains and the minimum inhibitory concentration (MIC) against *Pseudomonas aeruginosa* and *Streptococcus pneumoniae* was found to be 25 μg/mL. Our results confirm that plant endophytes are rich in biodiversity and contain important resource of many uncultured microorganisms.

## Introduction

Endophytes, including endophytic bacteria and endophytic fungi, are microorganisms that asymptomatically live in different healthy tissues of plants during the whole or part of their lifetime (Promputtha et al., 2005). Compared with the external dynamic environment of plants, a relatively stable environment can be obtained for microbial population adapted to endogenous life from the interior of the plant (Farrar et al., 2015; Siqueira et al., 2016). There are about 300,000 terrestrial host plant species distributed in temperate regions and tropical rain forests, each of which has one or more endophytes, and the biodiversity of these naturally occurring endophytes is very great (Jia et al., 2016; Doilom et al., 2017; Liu et al., 2017). Accumulating evidence has demonstrated that endophytes are hyperdiverse and abundant (Rodriguez et al., 2009; Zimmerman and Vitousek, 2012; Liu et al., 2017; Afzal et al., 2019). All plant species, such as angiosperms, non-vascular plants, conifers and ferns, are considered to be symbiotic with endophyte (Rosenblueth and Martinez-Romero, 2006; Rodriguez et al., 2009; Venugopalan and Srivastava, 2015). Studies have shown that endophytes provide some advantages to their host, including protection against insects/pests and pathogens (Rosenblueth and Martinez-Romero, 2006; Siqueira et al., 2011; Deshmukh et al., 2014; Zhang et al., 2016), conferring their host plant with an ability to adapt to stresses such as tolerance to heat, metals, drought, disease, herbivory and salinity, and also promote growth via plant hormone biosynthesis and nutrient acquisition (Rosenblueth and Martinez-Romero, 2006; Rodriguez et al., 2009; Venugopalan and Srivastava, 2015).

Although endophytes have been studied for a long time, they were found to be able to mimic the secondary metabolite repertoire of host plants only in the last two decades (Venugopalan and Srivastava, 2015). Endophytes can produce biologically active compounds, which are very important for increasing the adaptability of endophytes and their host plants, including tolerance to biotic and abiotic stresses (Rai et al., 2014; Golinska et al., 2015). More importantly, many compounds with unique structures and highly biologically active secondary metabolites were isolated from endophytes. An increasing number of novel compounds are being isolated from endophytic fungi and actinomycetes, including alkaloids, peptides, steroids, terpenoids, quinones, flavonoids, aliphatic compounds, and phenols (Strobel and Daisy, 2003; Golinska et al., 2015; Deshmukh et al., 2018). These compounds have been proved to have the biological activities previously shown, including antimicrobial, antiviral, larvicidal, antimalarial, anti-tumor and antidiabetic (Strobel and Daisy, 2003; Golinska et al., 2015; Matsumoto and Takahashi, 2017). Therefore, isolation of endophytes from medicinal plants may provide new opportunities to discover diverse species and natural products using for medicine, agriculture, and industry (Aly et al., 2011; Ding et al., 2013; Golinska et al., 2015; Jeewon et al., 2019; Bibi et al., 2020).

*Angelica sinensis* is a perennial herb of the Umbelliferae family and is widely planted in the western part of China. Radix *Angelica sinensis* (RAS) named as “*Danggui*” in China is one of the most important traditional Chinese medicine (Zhang et al., 2014, 2016). RAS is used to act on uterine smooth muscle for the treatment of irregular menstruation, to improve coronary circulation and atherosclerosis for the treatment of cardiovascular diseases (Zhang et al., 2013). Phthalides compound, organic acids and their esters and polysaccharides have been shown to be major chemical constituents associated with RAS’s high potency of pharmaceutical activity. In addition, the extracts of *Angelica* also have other pharmacological actions including radiation resistance, anti-inflammatory analgesia, anti-oxidation, anti-aging, and anti-psoriasis (Armstrong and Peterson, 2002; Bandara et al., 2006). Therefore, RAS is accepted by the ministry of health of China for use in health food raw materials, such as a daily tonic; furthermore, *A*. *sinensis* can be described as an edible Chinese traditional herb with a medicinal function (Tang, 2002).

As a traditional Chinese medicine material, *A*. *sinensis* is well documented for its various medicinal attributes and is clinically widely used. In view of the medicinal value of the host, in this study, we selected *A*. *sinensis* as a source plant to examine the diversity of the endophytic community with 20 different media. We also studied the diversity of endophytes in the seeds of *A*. *sinensis* using high-throughput sequencing. Finally, endophytes were isolated from healthy leaves, stems, roots and seeds of *A*. *sinensis*, and the metabolites of endophytic strains were evaluated with six different methods for their antioxidant activity and the paper disc diffusion method for antimicrobial activities. Our research will aid in the search for new and effective biologically active ingredients from endophytes associated with this medicinal plant and pave the way for future approaches to examining medicinal plants for their endophytes and systematically studying product isolation and characterization of bioactive compounds.

## Materials and Methods

### Plant Materials

Healthy *A*. *sinensis* plants were collected from Min county, Gansu Province in September 2015. Seeds were obtained from Zhang county, Gansu Province, China in October 2015. All samples were placed in ice boxes and immediately sent to the laboratory for further research.

### Chemicals and Reagents

The reference standard of rutin and gallic acid were purchased from Aladdin (purity > 98%, Shanghai, China). The 1,1 diphenyl 2-bitter hydrazine (purity **>** 97%) were purchased from Tokyo Chemical Industry. All other analytical chemicals were obtained from Kebaite Technology (Lanzhou, China).

### Tested Strains

Two strains of gram-negative bacteria (*Escherichia coli*, *Pseudomonas aeruginosa*) and three strains of gram-positive bacteria (*Streptococcus pneumoniae*, *Bacillus subtilis*, and *Staphylococcus aureus*) were used. These tested microbes were kindly provided by school of Basic Medical Sciences, Lanzhou University in China.

### Separation and Purification of *A*. *sinensis* Endophytes

The seed, leaves, stems and roots of fresh *A*. *sinensis* were rinsed thoroughly with running water for 10 min. They were soaked in 75% ethanol for 45 s. Then soaked in 2% NaClO for 4, 5, and 6 min, respectively, then the surface sterilizing agents were removed by rinsing 6 times with sterile distilled water. The last rinse was collected to evaluate the effect of sterilization (Zhang et al., 2016, 2017b). The surface sterilized plant samples were aseptically processed in an ice bath and plated on 20 different media shown in Table 1. As described Grum-Grzhimaylo et al. (2016), emerged fungal isolates were purified and pre-identified by morphological characters. To avoid redundancy, in few cases of joint isolations from different media, the co-isolated strains were discarded down to a minimum number of morphotypes (Grum-Grzhimaylo et al., 2016). All endophyte strains were kept in store at 4 °C and selected for further study.

**TABLE 1:** Twenty different media for endophyte isolation.

No	Medium	Ingredient
I	PDA	Potato 200 g, Dextrose 20 g, 15 g agar 1000 mL distilled water, pH 7.2
II	1/100 PDA	100 times diluted media I
III	Rose Bengal medium	5 g peptone, 10 g glucose, 1 g potassium dihydrogen phosphate, 0.5 g magnesium sulfate, 15 g agar, 0.03 g Rose Bengal, 0.1 g chloramphenicol, 1000 mL distilled water, pH 7.2 (Zhang et al., 2017a)
IV	1/100 Rose Bengal medium	100 times diluted media III
V	Czapek–Dox Medium	2 g NaNO_3_, 0.1 g K_2_HPO_4_, 0.5 g KCl, 0.5 g MgSO_4_, 0.01 g FeSO_4_, 30 g sucrose, 15 g agar and 1000 mL distilled water
VI	1/100 Czapek–Dox Medium	100 times diluted media V
VII	Yeast extract medium	10 g yeast extract, 20 g sucrose, 15 g agar 1000 mL distilled water, pH 7.2
VIII	1/100 Yeast extract medium	100 times diluted media VII
IX	Yam medium	Yam 200 g, Dextrose 20 g, 15 g agar 1000 mL distilled water, pH 7.2
X	1/100 Yam medium	100 times diluted media IX
IX	Carrot medium	Carrot 200 g, Dextrose 20 g, 15 g agar 1000 mL distilled water, pH 7.2
XII	1/100 Carrot medium	100 times diluted media IX
XIII	Lily medium	Lily 200 g, Dextrose 20 g, 15 g agar 1000 mL distilled water, pH 7.2
XIV	1/100 Lily medium	100 times diluted medium XIII
XV	Sweet potato medium	Sweet potato 200 g, 20 g Dextrose, 15 g agar 1000 mL distilled water, pH 7.2
XVI	1/100 Sweet potato medium	100 times diluted medium XV
XVII	beef extract peptone medium	5 g beef extract, 10 g peptone, 15 g agar, 5 g NaCl, 1000 mL distilled water, pH 7.2 (Zhang et al., 2017a)
XVIII	1/100 beef extract peptone medium	100 times diluted medium XVII
XIX	Multi-vitamins medium	0.4 g yeast extract, 4 g Dextrose, 3.75 mg multi-vitamins, 1 mL/L trace salt, 15 g agar, 1000 mL distilled water
XX	1/100 multi-vitamins medium	100 times diluted medium XIX
PS	Multi-vitamins	1 mg vitamin B_1_, 1 mg vitamin B_6_, 1 mg vitamin B_2_, 1 mg Nicotinic acid, 1 mg Phenylalanine, 1 mg vitamin H, 0.3 mg Alanin
	Trace salt	0.2 g MgSO_4_⋅7H_2_0, 0.1 g MnCl_2_⋅4H_2_0, 0.1 g ZnSO_4_⋅7H_2_0, 100 mL water

### Identification Based on Morphology and DNA Sequence Data

Morphological observation combined with molecular biology identification are widely used for taxonomic studies of strains (Zheng et al., 2016). The isolated fungi were grown on PDA medium and bacteria were grown on beef extract-peptone medium. Characterization of cell morphology included their mycelium color, pigment, morphology of spore producing structure, presence or absence of sporulation and spore morphology, and were described and surveyed using a microscope (Motic E220, China).

Ezup Column Genome DNA Extracting Kit (Sangon Biotech, Shanghai, China) was used to extract genomic DNA from high activity strains according to the manufacturer’s protocol. The 16S rDNA from the extracted DNA was amplified by PCR using primer 27F (5′-AGAGTTTGATCC TGGCTCAG-3′) and primer 338R (5′-TGCTGCCTCCCGTAGGAGT3′) (Zhang et al., 2017a); Moreover, 18S rDNA from the extracted DNA was amplified by PCR using primer EF3 (5′-TCC TCTAAATGACCAAGTTT-3′) and primer EF4 (5′-GGAAGGGRTGTATTTATTAG-3′).

Polymerase chain reaction (PCR) were performed using our previously reported method (Zhang et al., 2017b). Reaction mixtures contained 5 μL 10 × PCR buffer, 4 μL 2.5 mmol/L dNTPs, 1 μL 5 μmol/L forward primer, 1 μL 5 μmol/L reverse primer, 0.5 μL Taq enzyme, 1 μL Template DNA and was add ddH_2_O to 50 μL. The thermal cycling conditions were: denaturation at 94°C for 3 min, followed by 32 cycles of denaturation at 94°C for 30 s, annealing at 56°C for 30 s and an extension at 72°C for 50 s. At the end of the cycles, the reaction mixture was kept at 72°C for 7 min and then cooled to 4°C (Gutell and Fox, 1988). The gel electrophoresis (1%) was used to detect PCR products and the result were analyzed by Sangon Biotech (Shanghai, China).

The 16S rDNA or 18S rRNA gene sequences were subjected to BLAST search against the GenBank database. The sequences determined and reference sequences downloaded from GenBank were aligned using multiple-sequence alignment software CLUSTAL X version 1.81. Phylogenetic trees were constructed with the molecular evolutionary genetics analysis software MEGA 5.0 program.

### Diversity of Endosphere of Seeds in *A. sinensis*

According to the instruction manual, E.Z.N.A.DNAKit (Omega Bio-tek, Norcross, GA, United States) was used to extract microbial DNA from the seeds of *A*. *sinensis*, furthermore, determination of DNA concentration and purification were performed by using NanoDrop 2000 UV-vis spectrophotometer (Thermo Fisher Scientific, Wilmington, United States), and the gel electrophoresis (1%) was used to evaluate the quality of extracted DNA (Zheng et al., 2018). The 27F_338R hypervariable (V1-V2) regions of the bacteria 16S rRNA gene were amplified with primers 27F (5′-AGAGTTTGATCCTGGCTCAG-3′) and 338R (5′-TGCTGCCTCCCGTAGGAGT-3′) (Ravel et al., 2011) by PCR using a GeneAmp 9700 PCR system (ABI, United States). The TS1F_ITS2R hypervariable regions of the fungi 18S rRNA gene were amplified with primers ITS1F (5′-CTTGGTCATTTAGAGGAAGTAA-3′) and ITS2R (5′-GCTGCGTTCTTCATCGATGC −3′) by PCR. PCR reactions were performed using previous method (Chen et al., 2018) as follows: denaturation at 95°C for 3 min, followed by 27 cycles of denaturation at 95°C for 30 s, annealing at 56°C for 30 s and extension at 72°C for 45 s, and a final elongation at 72°C for 10 min (Chen et al., 2018). 2% agarose gel was used to extract the resultant PCR products, and further AxyPrep DNA Gel Extraction Kit (Axygen Biosciences, Union City, CA, United States) was used to purify and QuantiFluor-ST (Promega, United States) was used to quantify according to the manufacturer’s protocol (Chen et al., 2018). According to Majorbio Bio-Pharm Technology Co., Ltd. Standard protocol (Shanghai, China), Sequencing and analysis of the purified amplicons was performed on an Illumina MiSeq platform (Illumina, San Diego, CA, United States).

The Illumina reads of raw fastq files were demultiplexed and quality-filtered using Trimmomatic v0.36 (Bolger et al., 2014). Overlapping paired-end reads were merged using FLASH (Magoc and Salzberg, 2011) according to specific criteria as reported by Zhang et al. (2018). Chimeric sequences were identified and removed using UCHIME (Edgar et al., 2011). Sequences with 97% sequence similarity were clustered into Operational Taxonomic Units (OTUs) using UPARSE (version 7.1; Edgar, 2013)^1^. The taxonomy assignment of each OTU sequence was analyzed using the RDP Classifier algorithm^2^ against the Silva 16S rRNA database and Unite ITS fungi with a confidence threshold of 70% (Hao et al., 2017). The reference database used was Silva (Release 126)^3^. Rarefaction curves and alpha diversity containing the Chao1 index, ACE index, Shannon estimator and Simpson estimator were determined as described previously (Chen et al., 2017).

### Preparation of Endophytic Metabolite Extraction

Each fungal strain was inoculated into 200 mL potato dextrose broth (PDB) medium at 200 rpm on a rotary shaker at 28°C for 7–10 days. The fungal broth was filtered using Whatman No. 5 filter paper, and then the protein in filtrate was removed by the sevage method (Kusaikin et al., 2010). The mycelia was dried and then extracted twice with 95% ethyl alcohol (1:2/V:V) by circumfluence extraction (Cui et al., 2015). The bacteria was inoculated in beef extract-peptone medium at 200 rpm on a rotary shaker at 37°C for 48–72 h. All the extracting solution was concentrated in a rotary evaporator at 60°C and further was removed by evaporation under 40°C.

### Antioxidant Activity Assay

#### DPPH Radical Scavenging Activity Assay

The DPPH radical scavenging activity of endophytic metabolite extracts was measured according to the method of Du et al. (2013). 2.0 mL of different concentrations of the test sample (0.02, 0.04, 0.06, 0.08, and 0.1 mg/mL) which was added to a tube containing 2 mL of DPPH solution (0.05 mg/mL in ethanol). The reaction mixture was shaken well and incubated in the dark for 30 min at room temperature. Then, absorbance was measured at 517 nm. In this study, distilled water (negative) and ascorbic acid (positive controls) were used and the absorbance at 517 nm was measured. The following formula is used to calculate DPPH free radical scavenging activity (Du et al., 2013):

Scavenging rate (%) = (1–A_*x*__/_A_0_) × 100%

Where A_0_ is the absorbance value of the control, A_*x*_ is the absorbance value of the samples and ascorbic acid. IC_50_ is the amount of antioxidant necessary to decrease by 50% the initial DPPH concentration.

#### Hydroxyl Radical Scavenging Activity Assay

The hydroxyl radical scavenging activity of the endophytic metabolite extracts was measured following a method by Smirnoff and Cumbes (1989). 2.0 mL of different concentrations of the tested sample (0.02, 0.04, 0.06, 0.08, and 0.1 mg/mL) was added into a tube containing 1.0 mL FeSO_4_ (6.0 mmol/L), salicylic acid-ethanol (6.0 mmol/L) and 0.1% H_2_O_2_ reaction mixture, and cultured in a 37°C constant temperature water bath for 30 min. The absorbance of the reaction mixture was read at 510 nm. The hydroxyl radical-scavenging rate was calculated using the following formula:

Hydroxyl radical-scavenging rate (%) = [1 – (A_1_− A_2_)/A_0_] × 100% (Du et al., 2013)

In the above formula, A_0_ represents OD_510_ of the blank control, A_1_ represents OD_510_ of the sample and positive (ascorbic acid), and A_2_ represents OD_510_ of the sample and the positive control with distilled water containing 2 mL instead of H_2_O_2_ aqueous (Du et al., 2013).

#### Ferric Cyanide Reducing Power Assay

Ferric cyanide reducing power (FRAP) of the endophytic metabolite extracts was determined by the method of Wang et al. (2012). Reaction mixtures were prepared by adding 2.5 mL of phosphate buffer (0.2 M, pH 6.6), 2.5 mL potassium ferricyanide (1%) and 2.5 mL varying concentrations (0.02, 0.04, 0.06, 0.08, and 0.1 mg/mL) of tested samples and incubating reaction mixtures at 50°C for 30 min, allowing to cool at room temperature (28°C), and adding 2.5 mL of 10% TCA (Trichloroacetic acid) to each reaction mixture, followed by centrifugation at 5000 rpm for 5 min. Five milliliter of the supernatant was added to 4 mL of distilled water and 1 mL of 1.0% FeCl_3_ in a test tube, and the absorbance was measured at 700 nm after reacting at room temperature for 10 min. Ascorbic acid solution was used as a standard.

#### Total Antioxidant Capacity Assay

The total antioxidant capacity of the endophytic metabolite extracts was determined using the phosphomolybdate method as described by Naima et al. (2012). 0.5 mL of different concentrations (0.02, 0.04, 0.06, 0.08, and 0.1 mg/mL) of tested sample and 5.0 mL of reagent solution (0.6 M sulfuric acid, 28 mM sodium phosphate, 4 mM ammonium molybdate) were mixed in a tube and placed at 95°C for 90 min. After the mixture cooled to room temperature, the absorbance of 765 nm was noted as A_*x*_. 5.0 mL blank control containing reagent solution and the appropriate volume of solvent was incubated under the same conditions. Ascorbic acid as a standard control was noted as A_0_. The total antioxidant capacity was calculated by the following formula:

Total antioxidant capacity (%) = (A_0_ − A_*x*_)/A_0_ × 100%.

#### Hydrogen Peroxide Radical (H_2_O_2_) Scavenging Assay

The hydrogen peroxide scavenging of the endophytic metabolite extracts was determined using the method previously reported by Ruch et al. (1989). The reaction mixture was prepared by adding hydrogen peroxide solution (2 mM) to 50 mM phosphate buffer (pH 7.4). 0.5 ml of different concentrations (0.02, 0.04, 0.06, 0.08, and 0.1 mg/mL) of tested sample was placed into the test tube and diluted with phosphate buffer to 2.0 mL. Subsequently, 3.0 mL hydrogen peroxide solution was added to the mixture, and the absorbance of 230 nm was measured after 10 min. The hydrogen peroxide scavenging activities were calculated by the following formula:

Hydrogen peroxide scavenging activities (%) = (A_0_−A_*x*_)/A_0_) × 100%,

Where A_0_ and A_*x*_ was the absorbance values of ascorbic acid and the samples, respectively.

#### Superoxide Anion Radical Scavenging Assay

The superoxide anion scavenging activity of the endophytic metabolite extracts was measured by following the method of Nishikimi et al. (1972) and Kaur and Arora (2010). One milliliter nitroblue tetrazolium (NBT) solution (156 μM NBT in 100 mM phosphate buffer, pH 8.0), 1 mL NADH solution (468 μM in 100 mM phosphate buffer, pH8.0) and 1 mL of different concentrations (0.02, 0.04, 0.06, 0.08, and 0.1 mg/mL) of the tested sample were mixed. 60 μM of phenazine methyl sulfate (PMS) was dissolved in pH 8.0, 100 mM phosphate buffer, and further 100 μl PMS was added to start the reaction. After the reaction was carried out at 25°C for 5 min, the absorbance values of 560 nm were measured. The capability of scavenging super oxide radicals was calculated using the following formula (Kaur and Arora, 2010):

Capability of scavenging super oxide radicals (%) = (A_0_−A_*x*_)/A_0_ × 100%.

Where A_0_ and A_*x*_ was the absorbance of the positive control (ascorbic acid) and the samples, respectively. The solution containing NBT, NADH, and PMS without the sample was used as a blank.

### Determination of Total Flavonoid Content

The total flavonoid content of the endophytic metabolite extracts was determined using the method previously reported by Park et al. (2008). 1.0 mL of tested sample (0.1 mg/mL) was mixed with 2 mL of distilled water, after which 0.5 mL NaNO_2_ (0.5 M) and 0.5 mL Al(NO_3_)_3_ (0.3 M) solution were added to the mixture. After 6 min, 0.5 mL NaOH solution (1.0 M) was added to the mixture and cultured for 30 min at room temperature. Absorbance of 510 nm was determined with rutin as a standard compound.

### Determination of Total Phenolic Content

Total phenolic acid content was determined by the Folin-phenol method (Kim et al., 2003). One milliliter of tested sample (0.1 mg/mL) and 1 mL Folin-ciocalteu reagent were added to a flask and mixed thoroughly. After 3 min, 3 mL of sodium carbonate (2%) was added and the mixture incubated at 23°C for 90 min with intermittent shaking. The absorbance was measured at 750 nm versus water blank with gallic acid as a standard compound.

### Determination of Antibacterial Activity

Antimicrobial activity of the secondary metabolite of endophytes was evaluated using the paper disc diffusion method, as described by Doughari (2006). For determination of antibacterial activity, all bacterial strains were suspended in nutrient broth and incubated at 37°C, after which they were adjusted to approximately 10^5^ colony forming units/mL with sterile saline (Clinical and Laboratory Standards Institute, 2002) and 200 μL of suspensions was spread over nutrient agar (beef-oxoid) plates (diameter: 9 cm). Extracts were dissolved in 10% aqueous dimethylsulfoxide (DMSO) and sterilized by filtration through a 0.22 μm membrane filter. Under aseptic conditions, empty sterilized discs (diameter: 6 mm) were impregnated with 10 μL of extract dilutions (10 mg/mL) and placed on the previously-seeded agar surface. Paper discs impregnated with 10 μL of penicillin (5 mg/mL) and 10 μL of DMSO were used as positive and negative controls, respectively. All plates were incubated at 37°C for 18 h. Antimicrobial activity was determined by the apparent zone clear zones of inhibition around the paper discs. For each extract, three replicates were performed against each organism (Wang et al., 2012).

### Determination of the Minimum Inhibitory Concentration (MIC)

The minimum inhibitory concentration (MIC) of the secondary metabolites of the endophytes was determined by the paper diffusion method, as described by Shafi (1975). Ten microliter of varying concentrations of the extracts (0.01, 0.05, 0.5, 1.0, 2.0, 4.0, 6.0, 8.0, and 10.0 mg/mL) were placed on the agar surface previously seeded using the above-mentioned method. Paper discs impregnated with 10 μL of a solution of 5 mg/mL of penicillin as a standard antimicrobial were used for comparison. All petri dishes were sealed with sterile laboratory parafilm to avoid eventual evaporation of the test samples. Inoculated plates were incubated at 37°C for 18 h. Inhibition of bacterial growth on the plates containing the test samples was determined by comparison with growth on the blank control plates. The MIC of each test sample was determined as the lowest concentration to completely inhibit the growth of each organism (Shafi, 1975; Delaquis et al., 2002). The MIC was repeated for each of the test organisms in triplicate (Shafi, 1975).

### Data Analysis

All experimental data are expressed as mean ± SD from three separate observations. The data analyzed by one-way analysis of variance (ANOVA) followed by Duncan’s multiple range tests using the SPSS19.0 software. *P* < 0.05 was used to define statistically significant differences between the control and experimental groups.

## Results

### Isolation and Identification of Endophytes

**FIGURE 1:**
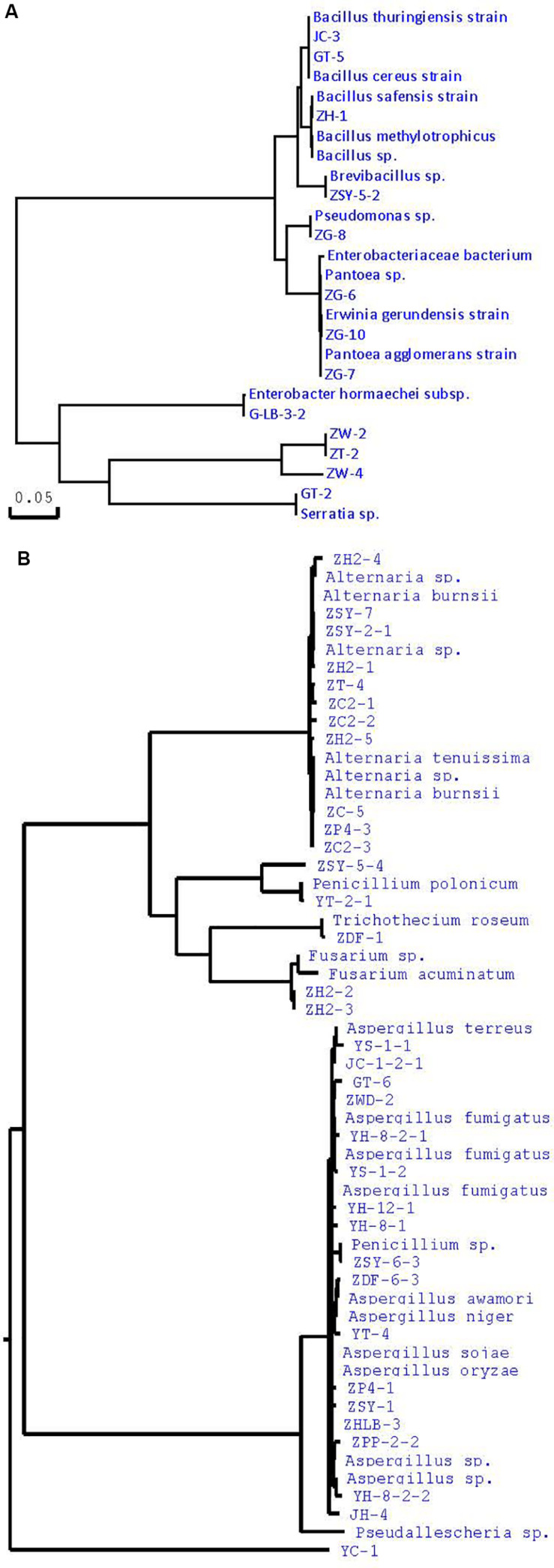
Phylogenetic tree of partial sequence of endophytes from *A*. *sinensis* (**A**, bacteria; **B**, fungi).

Many studies have confirmed that endophytes have obvious distribution diversity in different tissue of host plants (Kumar and Hyde, 2004; Rosenblueth and Martínez-Romero, 2004; Promputtha et al., 2007; Jeewon et al., 2013). Therefore, in this study, different tissue of *A*. *sinensis*, including roots, stems, leaves, and seeds, are used for the isolation of endophytes. A large number of endophytes were isolated from different tissues of *A*. *sinensis*, including leaves, stems, roots and seeds, all of which showed that endophytes are very abundant and diverse in *A*. *sinensis*. After inoculation and culturing for more than 1 week, 226 endophyte strains with distinct morphology were isolated from the roots, stems, leaves and seeds of *A*. *sinensis* samples. All tissues showed a variation in the colonization frequency of endophytes. Among the tissues sampled, maximum colonization frequency was found in seed (29%), followed by leaf (26%) and lastly in stem and root with 23 and 22%, respectively. The molecular identification of 47 typical strains combined with morphology is shown in Table 2. The maximum nucleotide identity of the 16S DNA sequences of endophyte compared with those available in GenBank ranged from 99 to 100% (Table 2; Wȩżowicz et al., 2014). The 16S rDNA-based phylogenetic tree provides a more detailed synopsis of the relationship between the different species of endophytes obtained from different parts of each plant (Kusari et al., 2013; Figure 1A). The tree shows two major groups, which comprise 13 isolates belonging to *Firmicutes* and *Proteobacteria*, and includes *Brevibacillus* sp., *Pantoea* sp., *Erwinia* sp., *Enterobacter* sp., *Pseudomonas* sp., *Bacillus*, and *Serratia* sp. As shown in Figure 1B, a phylogenetic tree was built from 18S rDNA sequences of endophytic fungi. The tree shows two major groups, which comprises 34 isolates belonging to *Ascomycota* and *Deuteromycetes*, and corresponds to *Penicillium* sp., *Fusarium* sp., *Trichothecium* sp., *Aspergillus* sp., and *Alternaria* sp.

**TABLE 2:** Endophytes isolated from different tissues of *A. sinensis.*

Endophytes	Genus	Maximum identity (%)	Accession
ZC-5	*Alternaria burnsii*	100%	MT487761
ZC2-1	*Alternaria alternata*	100%	MT487762
ZC2-2	*Alternaria* sp.	99%	MT487763
ZC2-3	*Alternaria tenuissima*	100%	MT487764
ZHLB-3	*Aspergillus fumigatus*	100%	MT487765
ZDF-6-3	*Aspergillus awamori*	99%	MT487766
ZG-6	*Pantoea* sp.	99%	MT492013
ZG-10	*Erwinia gerundensis*	99%	MT492014
ZG-8	*Pseudomonas* sp.	100%	MT492015
ZG-7	*Pantoea agglomerans*	99%	MT492016
ZG-LB-3-2	*Enterobacter cloacae*	100%	MT505514
ZDF-1	*Trichothecium roseum*	99%	MT487767
ZH-1	*Bacillus safensis*	100%	MT492017
ZH2-1	*Alternaria burnsii*	100%	MT488301
ZH2-2	*Fusarium* sp.	100%	MT563084
ZH2-3	*Fusarium acuminatum*	100%	MT487768
ZH2-4	*Alternaria* sp.	99%	MT498091
ZH2-5	*Alternaria alternata*	99%	MT487769
ZP4-1	*Aspergillus fumigatus*	99%	MT487770
ZP4-3	*Alternaria tenuissima*	100%	MT487771
ZPP-2-2	*Aspergillus sojae*	99%	MT487772
ZSY-1	*Aspergillus fumigatus*	100%	MT487773
ZSY-2-1	*Alternaria alternata*	100%	MT487774
ZSY-5-2	*Brevibacillus* sp.	100%	MT492018
ZSY-5-4	*Aspergillus fumigatus*	100%	MT487775
ZSY-6-3	*Penicillium* sp.	99%	MT487776
ZSY-7	*Alternaria* sp.	99%	MT487777
ZT-2	*Bacillus methylotrophicus*	100%	MT492019
ZT-4	*Alternaria* sp.	100%	MT487778
ZW-2	*Bacillus* sp.	100%	MT492020
ZW-4	*Enterobacteriaceae bacterium*	99%	MT492021
ZWD-2	*Aspergillus fumigatus*	99%	MT487779
YC-1	*Pseudallescheria* sp.	100%	MT563085
YH-8-1	*Aspergillus fumigatus*	99%	MT487780
YH-8-2-1	*Aspergillus* sp.	99%	MT487781
YH-8-2-2	*Aspergillus* sp.	100%	MT487782
YH-12-1	*Aspergillus oryzae*	100%	MT487783
YS-1-1	*Aspergillus terreus*	100%	MT487784
YS-1-2	*Aspergillus fumigatus*	99%	MT487785
YT-2-1	*Penicillium polonicum*	100%	MT487786
YT-4	*Aspergillus niger*	99%	MT487787
JC-3	*Bacillus thuringiensis*	100%	MT492022
JH-4	*Aspergillus* sp.	99%	MT487788
JC-1-2-1	*Aspergillus terreus*	100%	MT487789
GT-2	*Serratia* sp.	100%	MT505513
GT-5	*Bacillus cereus*	100%	MT492023
GT-6	*Aspergillus fumigatus*	99%	MT487790

### Biodiversity and Abundance of Bacteria and Fungi With Non-culture

A total of 252,400 fungi sequences and 362,404 bacterial sequences were obtained from 4 seed samples of *A*. *sinensis*. The length of these valid sequences was approximately 280–340 bp for bacteria and 220–260 for fungi, accounting for 99% of the total valid sequences. The mean length of these valid sequences was nearly 290 bp for bacteria and 250 bp for fungi. The rarefaction curves of sequence data showed clear asymptotes in the number of observed species, demonstrating that bacterial and fungal communities from each seed sample of *A*. *sinensis* were well-covered by pyrosequencing (Supplementary Figure S1).

Using a 97% similarities sequence cutoff value, the valid sequences were further clustered into 152 OTU for bacteria and 83 OTU for fungi. Furthermore, a Community Diversity index, including Ace, Sobs and Chao, and a Community richness estimator, including Shannon and Simpson, were further analyzed (Supplementary Table S1). No significant differences between the Community Diversity index and Community richness estimator could be observed for bacteria and fungi, suggesting that the difference in the diversity of bacteria and fungi, in the *A*. *sinensis* seeds, is not obvious and that as previously reported, bacterial and fungal diversity in the rhizosphere around the plants are similar (Xiao et al., 2013). Moreover, the coverage shown in Supplementary Table S1 was almost the same (from 99.95 to 100.0%), which was consistent with the demonstration of the rarefaction curve (Supplementary Table S1 and Supplementary Figure S1; Wu et al., 2016).

**FIGURE 2:**
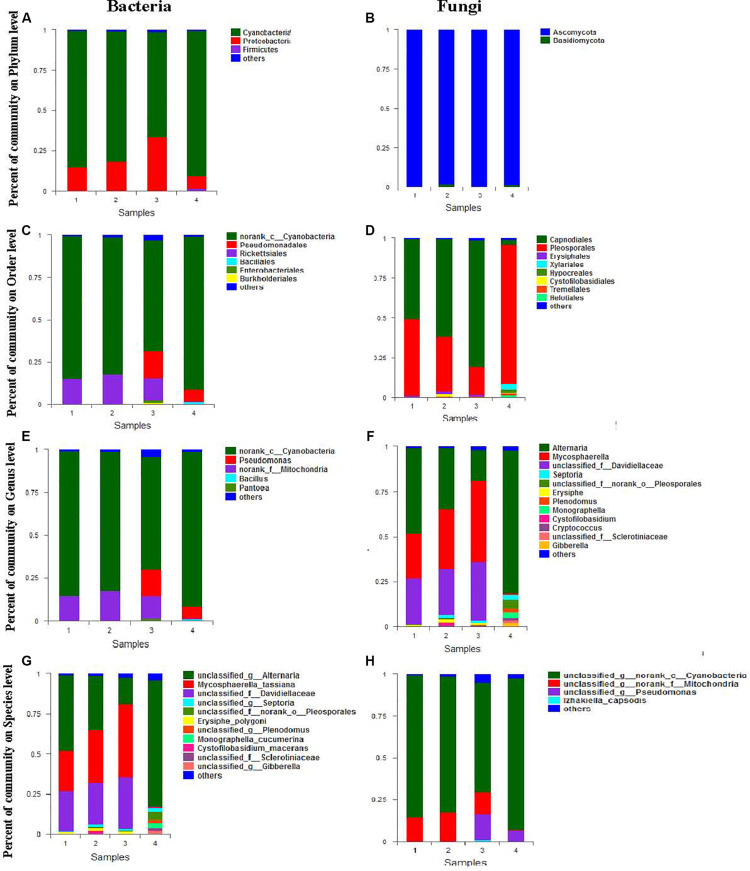
Mean relative abundance of the abundant phyla, orders, genera, and species in bacteria **(A,C,E,G)** and fungi **(B,D,F,H)**.

The relative ratio of bacteria to fungi composition at the phylum level is shown in Figure 2. As shown in Figure 2A, bacterial sequences were classified into 7 phyla. the main phyla in the four biological samples were *Cyanobacteria* (65.45–90.4%), *Proteobacteria* (7.64–33.23%), and *Firmicutes* (0.04–1.46%). The relatively low abundance (0.24–1.24%) of *Actinobacteria*, *Bacteroidetes*, *Microgenomates*, and *Saccharibacteria* was also detected in a most biosamples. As shown in Figure 2B, fungi from two phyla were detected, the main phylum in the four biosamples was *Ascomycota* (98.15–99.56%). The relatively low abundance (0.44–1.85%) of *Basidiomycota* was also detected in most biosamples.

The relative ratio of bacteria to fungi composition at the order level is shown in Figure 2. Relative abundance analysis showed 26 orders of bacteria and 17 orders of fungi from each seed sample of *A*. *sinensis*. As demonstrated in Figure 2C, the dominant bacteria order with abundance larger than 1% (the main order with abundance larger than 1%) in the four biosamples was *norank_c__Cyanobacteria* (65.45–90.4%), *Rickettsiales* (0.47–17.45%), *Pseudomonadales* (0.09–15.87%), *Enterobacteriales* (0.07–1.53%), and *Bacillales* (0.01–1.2%). The relatively low abundance (0–1.0%) of *Lactobacillales*, *Burkholderiales*, *Sphingobacteriales*, *Rhodospirillales*, *Rhizobiales Micrococcales*, *Cytophagales*, and *Streptomycetales* were also detected in most biosamples. As demonstrated in Figure 2D, the dominant fungi order with abundance larger than 1% (the main order with abundance larger than 1%) in the four biosamples was *Pleosporales* (17.34–87.57%), *Capnodiales* (3.28–79.6%), *Erysiphales* (0.00–1.67%), *Helotiales* (0.01–1.49%), and *Tremellales* (0.01–1.26%). The relatively low abundance (0–1.0%) of *Unclassified_c__Dothideomycetes*, *Cystofilobasidiales*, *Eurotiales*, *Dothideales*, *Xylariales*, and *Pleosporales* were also detected in most biosamples.

The relative ratio of bacteria to fungi composition at the genus level is shown in Figure 2. Relative abundance analysis showed that 88 genera of bacteria and 44 genera of fungi were found in each seed sample of *A*. *sinensis*. As demonstrated in Figure 2E, the dominant bacterial genus with abundance larger than 1% (the main order with abundance larger than 1%) in the four biosamples were *norank Cyanobacteria* (65.45–90.4%), *Rickettsiales* (0.47–17.45%), *Pseudomonadales* (0.09–15.87%), *Enterobacteriales* (0.07–1.53%), and *Bacillales* (0.01–1.2%). The relatively low abundance (0–1.0%) of *Lactobacillales*, *Burkholderiales*, *Sphingobacteriales*, *Rhodospirillales*, *Rhizobiales Micrococcales*, *Cytophagales*, and *Streptomycetales* were also detected in most biosamples. As demonstrated in Figure 2F, in the four biosamples, the dominant fungi order with abundance larger than 1% (the main order with abundance larger than 1%) were *Alternaria* (16.79–79.28%), *Mycosphaerella* (2.27–45.5%), *Unclassified Davidiellaceae* (0.12–32.46%), *Unclassified norank Pleosporales* (0.21–4.94%), *Monographella* (0.0–3.03%), *Plenodomus* (0.1–2.27%), *Septoria* (0.2–2.57%), *Gibberella* (0.01–2.16%), *Erysiphe* (0.78–1.67%), *Unclassified Sclerotiniaceae* (0.01–1.47%) and *Cystofilobasidium* (0.0–1.4%). The relatively low abundance (0–1.0%) of *Unclassified Capnodiales*, *Unclassified Dothideomycetes*, *Aspergillus*, *Cryptococcus*, *Stagonosporopsis*, *Stemphylium*, *Cladosporium*, *Trichothecium*, and *Mycosphaerella* was also detected in most biosamples.

The relative ratio of bacterial and fungal composition at species level is shown in Figure 2. Relative abundance analysis showed 125 species of bacteria and 60 species of fungi from each seed sample of *A*. *sinensis*. As demonstrated in Figure 2G, the dominant bacterial genera with an abundance higher than 1% (the main order with abundance larger than 1%) in the four biosamples were *Unclassified norank Cyanobacteria* (65.44–90.38%), *Unclassified norank Mitochondria* (0.47–17.44%), *Unclassified Pseudomonas* (0.01–15.22%), and *Izhakiella capsodis* (0–1.15%). The relatively low abundance (0–1.0%) of *Unclassified Asaia*, *Uncultured bacterium Hymenobacter*, *Unclassified Oxalobacteraceae*, *Unclassified Massilia*, *Uncultured bacterium Variovorax*, *Ethylobacterium adhaesivum*, and *Methylobacterium* were also detected in most biosamples. As demonstrated in Figure 2H, the dominant fungal species with abundance larger than 1% (the main order with abundance larger than 1%) in the four biosamples were *Unclassified Alternaria* (16.74–79.26%), *Mycosphaerella_tassiana* (0.59–45.5%), *Unclassified Davidiellaceae* (0.12–32.46%), *Unclassified norank Pleosporales* (0.21–4.94%), *Monographella cucumerina* (0.0–3.03%), *Unclassified Plenodomus* (0.1–2.15%), *Unclassified Septoria* (0.2–2.57%), *Unclassified Gibberella* (0.01–2.16%), *Erysiphe polygoni* (0.78–1.67%), *Unclassified Sclerotiniaceae* (0.01–1.47%), and *Cystofilobasidium* (0.0–1.4%). The relatively low abundance (0–1.0%) of *Unclassified Capnodiales*, *Unclassified Dothideomycetes*, *Aspergillus*, *Cryptococcus*, *Stagonosporopsis*, *Stemphylium*, *Cladosporium*, *Trichothecium* and *Mycosphaerella*, *Unclassified Rachicladosporium*, *Unclassified Capnodiales*, *Unclassified Rachicladosporium*, *Unclassified Penicillium*, *Unclassified Cryptococcus*, *Unclassified Ascomycota*, and *Unclassified Capnodiales* were also detected in most biosamples.

### DPPH Free Radical Scavenging

In the current study, the antioxidant activity of 190 endophytic metabolite extracts was evaluated using the DPPH scavenging method. The primary results of the DPPH radical scavenging assay are shown in Table 3 (scavenging rates > 70%). As can be seen, scavenging rates between 0 and 70%, 70 and 80%, 80 and 90%, and 9 and 100% were found in 49 (46.23%), 13 (12.26%), 21 (19.81%), and 1 (0.94%) isolates, respectively (Table 3). The DPPH radical scavenging rate of ZP4-3 fermentation, JH-4 mycelia, JH-4 fermentation and GT-2 fermentation exceeded 85%, indicating significant antioxidant activity, with the highest values observed for ZP4-3 fermentation (95.21 ± 1.131%).

**TABLE 3:** Number of endophytes with different DPPH radical-scavenging rates.

DPPH radical scavenging	Number of endophytes				
	Seed	Leaf	Stem	Root	Total (%)
∼70%	18	11	8	12	46.23
∼80%	4	3	5	1	12.26
∼90%	8	4	4	5	19.81
∼100%	1	0	0	0	0.94

**FIGURE 3:**
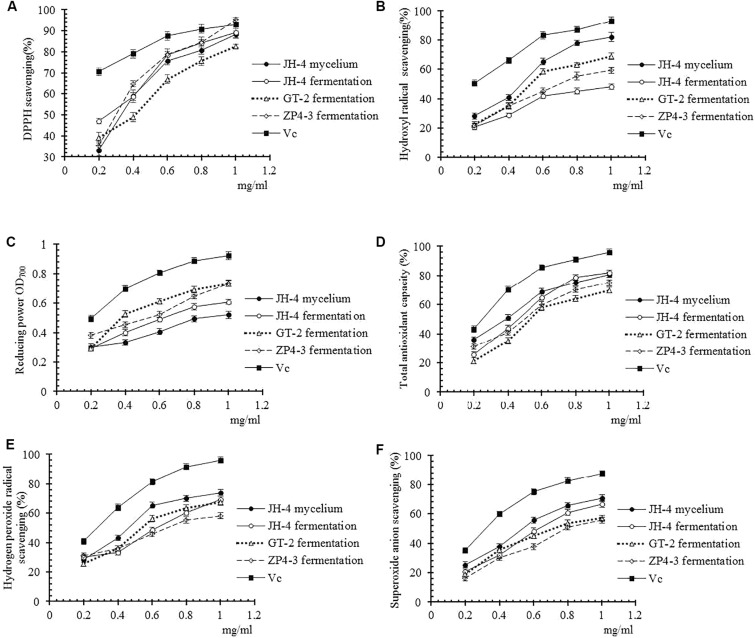
Antioxidant activities of different samples. **(A)** DPPH radical scavenging activity. **(B)** Hydroxyl radical scavenging activity. **(C)** Reducing power. **(D)** Total antioxidant capacity. **(E)** Hydrogen peroxide radical activity. **(F)** Superoxide anion scavenging activity.

Moreover, the antioxidant activities of these four samples were further determined using different methods at different concentrations. As shown in Figures 3A–F, our results showed that four tested samples exhibited antioxidant activity in a dose-dependent manner within a concentration range of 0.02–0.1 mg/mL. Similar to the standard V_*C*_, over a certain concentration range radical scavenging ability increased before reaching a plateau (Figures 3A–F). Furthermore, the IC_50_ of the four samples of DPPH radicals, hydroxyl radical, reducing power, total antioxidant capacity, hydrogen peroxide and superoxide anion are determined and summarized in Table 4.

**TABLE 4:** IC_50_ value from five antioxidant activity assays from four extracts of *A*. *sinensis*.

	IC_50_ value (mg extract per mL)				
	DPPH radical	Hydroxyl radical	Total antioxidant capacity	Hydrogen peroxide radicals	Superoxide anion
JH-4 mycelium	0.057 ± 0.002^a^	0.061 ± 0.002^d^	0.062 ± 0.004^c^	0.068 ± 0.002^d^	0.071 ± 0.006^c^
JH-4 fermentation	0.056 ± 0.006^a^	0.104 ± 0.006^a^	0.060 ± 0.003^c^	0.072 ± 0.009^c^	0.073 ± 0.004^c^
GT-2 fermentation	0.057 ± 0.002^a^	0.073 ± 0.003^c^	0.071 ± 0.008^a^	0.074 ± 0.008^b^	0.088 ± 0.007^b^
ZP4-3 fermentation	0.053 ± 0.002^a^	0.084 ± 0.007^b^	0.067 ± 0.002^b^	0.086 ± 0.003^a^	0.090 ± 0.003^a^
V_*C*_	0.054 ± 0.005^a^	0.053 ± 0.002^*e*^	0.053 ± 0.003^d^	0.052 ± 0.005^*e*^	0.056 ± 0.007^*e*^

*^*a*–*e*^significant differences between different groups (p < 0.05).*

As shown in Figure 3A and Table 4, the results show that no significant difference between the IC_50_ of the four samples of DPPH radicals (*P* > 0.05) (Table 4), indicating that the antioxidant activity of the tested samples was similar to the V_*C*_ standard. As shown in Figures 3B–F and Table 4, compared with Vc, the IC_50_ of the four samples for hydroxyl radical, reducing power, total antioxidant capacity, hydrogen peroxide and superoxide anion was significantly lower (*P* < 0.05). However, the IC_50_ of the JH-4 mycelium for the hydroxyl radical, hydrogen peroxide radicals and superoxide anion were significantly higher compared with the other three samples (*P* < 0.05). The JH-4 mycelium IC_50_ for DPPH radicals, hydroxyl radical, reducing power, total antioxidant capacity, hydrogen peroxide and superoxide anion was 0.057 ± 0.002, 0.061 ± 0.002, 0.062 ± 0.004, 0.068 ± 0.002, and 0.071 ± 0.006 mg/mL, respectively, all of which suggest that the antioxidant activity of JH-4 mycelia was the strongest.

### Estimation of Total Flavonoid and Total Phenol Content

**FIGURE 4:**
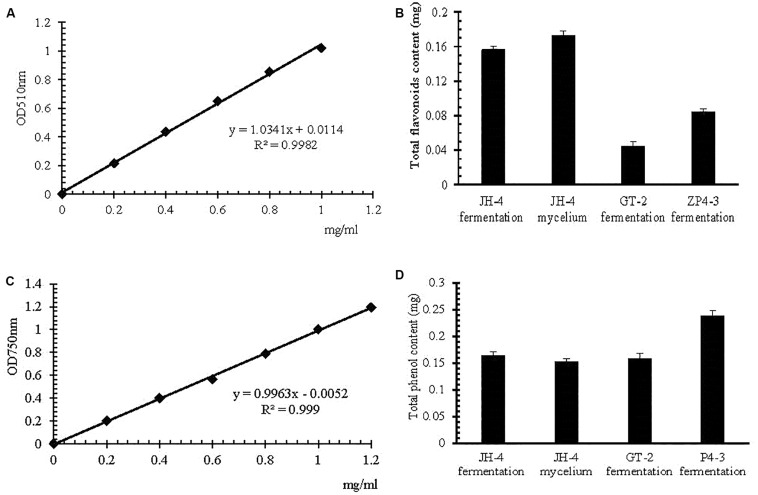
Estimation of total flavonoid and total phenol content. **(A)** Rutin standard curve. **(B)** Total flavonoid content. **(C)** Gallic acid standard curve. **(D)** Total phenol content.

The method of estimation of total flavonoid content and total phenol content was developed in the present study, the result of which is shown in Figure 4. The standard curve of the rutin standard and gallic acid show a good linearity (*R*^2^ > 0.99) over the concentration range of 0–1.2 mg/mL (Figures 4A,C). The results suggests that the method is applicable for the determination of total flavonoid content and total phenol content (Clinical and Laboratory Standards Institute, 2002). The total phenolic content in JH-4 fermentation, JH-4 mycelium, GT-2 fermentation and ZP4-3 fermentation were estimated to be 0.164 ± 0.008 mg/mL, 0.142 ± 0.009 mg/mL, 0.148 ± 0.004 mg/mL, and 0.229 ± 0.008 mg/mL, respectively (Figure 4B). The total flavonoid content in JH-4 fermentation, JH-4 mycelium, GT-2 fermentation and ZP4-3 fermentation were estimated to be 0.156 ± 0.004 mg/mL, 0.173 ± 0.003 mg/mL, 0.044 ± 0.004 mg/mL, and 0.084 ± 0.006 mg/mL, respectively, of which the highest total content of flavonoids was found in the JH- 4 mycelium, shown in Figure 4D.

### Determination of Antimicrobial Activity and MIC Assay

This study also aimed to obtain isolates with high antimicrobial activity from *A*. *sinensis*. We found that some sample of 190 crude extracts of endophytes from *A*. *sinensis* mostly displayed inhibitory activity against one or more of the five indicator bacteria. The primary screening results also showed that the mycelia extracts displayed higher antimicrobial activity than those of the filtrates. Furthermore, the MIC of four extracts (ZHLB-3 mycelium, ZWD-1 mycelium, YS-1-2 mycelium, and YH-12-1 mycelium) with high antimicrobial activity from the 190 endophytic metabolite extractions were determined, and the results are shown in Table 5. As we can see, YH-12-1 mycelium exhibited good inhibitory activity against the five test strains, including *Escherichia coli*, *Pseudomonas aeruginosa*, *Bacillus licheniformis*, *Staphylococcus aureus*, and *Streptococcus pneumoniae*. In particular, the MIC value against *P*. *aeruginosa* and *S*. *aureus* was 25 μg/mL, and MIC value against *B*. *licheniformis* was 30 μg/mL. Compared with the MIC of ampicillin against *E*. *coli* and *S*. *aureus* of 8 and 6 μg/mL, this strain showed very good potential for further research.

**TABLE 5:** Determination of Minimum inhibitory concentration (MIC) (μg/mL).

	*Escherichia coli*	*Pseudomonas aeruginosa*	*Bacillus licheniformis*	*Staphylococcus aureus*	*Streptococcus pneumoniae*
ZHLB-3 mycelium	> 50	30	>50	> 50	50
ZWD-1 mycelium	> 50	50	50	> 50	>50
YS-1-2 mycelium	> 50	30	>50	50	> 50
YH-12-1 mycelium	> 50	25	30	25	50

## Discussion

Endophytes are widely distributed in plant tissues as well as the interior of plant tissues. Endophytes with different morphologies and functions are isolated from various plant species in natural ecosystems (Tejesvi et al., 2011), and play an important role in host life cycles (Rodriguez et al., 2009; Liu et al., 2017). Western China, especially in Gansu Province, has a unique alpine climate and is a suitable place for *A*. *sinensis* planting. Some areas are called as “the homeland of Chinese *A*. *sinensis*,” and its production of artificially cultivated *A*. *sinensis* constitutes 70% of China’s total production (Zhang et al., 2016). Therefore, in the present study, fresh *A*. *sinensis* plants from Min County of Gansu province were used to isolate endophytes using a tissue homogenate method.

The biosphere is microbially rich. However, after nearly 200 years of microbiology discovery research, we still know very little about the microbial diversity around us (Epstein, 2013). Most microorganisms in the natural environment are difficult or impossible to obtain pure culture by traditional culture separation methods. Traditionally, microbiologists try to increase the separation of microorganisms by changing the nutrient composition and culture conditions of these classic media (Achtman and Wagner, 2008; Epstein, 2013). In this study, to isolate as many endophytes as possible and simulate the artificial environment as much as possible, 20 different media, including oligotrophic media, were used along with the conventionally diluted media. Generally, PDA medium is the most commonly used medium for the isolation of endophytes, in which, potato extract is the main natural complex component that affects the isolation of endophytes, and artificial medium cannot fully match. The potato as a tuber plant was used as a natural medium, therefore, other tuber plants such as lily, sweet potato, yam and carrot were also used as natural medium components for the separation of endophytes in here. However, the above natural medium is sufficiently nutritious, and rapid growth of dominant strains would inhibit the growth of other non-dominant strains, resulting in a decrease in the number and abundance of endophytes. Studies have shown that by reducing the nutrient concentration of traditional medium to prepare the oligotrophic state, and appropriately extending the incubation time, some microorganisms that cannot be separated from the classical medium can be obtained (Davis et al., 2005; Epstein, 2013). Therefore, a variety of oligotrophic media were prepared with 100-fold diluted natural medium and were used in the experiment to isolate the endophytes. The results showed that the separation efficiency of the 100-fold diluted natural medium was higher than that of the original medium, which 100-fold diluted yam medium was the highest.

With 20 different media used, the results showed there are many endophytes in *A*. *sinensis*, which have abundant biodiversity. Furthermore, 226 representative strains were isolated and distributed in at least 13 genera belonging to 4 phyla. For endophytic fungi, the most frequent genera were *Alternaria* sp. and *Aspergillus* sp. The results also agreed with other previous reports that members of a variety of common fungal genera, such as *Alternaria* sp., *Aspergillus* sp., *Penicillium* sp., and *Fusarium* sp., are usually isolated from a variety of plants around the world (Yang et al., 2013; Wȩżowicz et al., 2014). In addition, organ specificity of endophytes were observed (Kusari et al., 2013). For example, in the present study, *Serratia* sp. preferred to colonize the roots and *Pseudallescheria* sp. inhabited only the leaf. However, *Brevibacillus* sp., *Erwinia* sp., *Enterobacter* sp., and *Pseudomonas* sp. inhabited only the seed. Compared with the MiSeq sequencing method, a minority of endophytes were identified from *A*. *sinensis*. This indicates that there are still some defects in the traditional approaches. Therefore, different cultivation methods, such as diffusion box cultivation *in situ*, still need to be carried out in future.

We live in a world surrounded by microbes, the endophyte in a plant includes both the endogenous bacteria that are inherent to the plant and the inter-endogenous bacteria that coexist between the plants. As a class of microbes coexisting with host plants, endophytes have become an increasingly good resource for finding new active compounds. Plant seeds are not merely reproductive organs or important tools in agricultural production, they are also carriers of both pathogens and beneficial endophytes, particularly, inherent and non-invasive characteristic endophytes in host plant. However, in comparison to the well-studied endophytic fungi of roots, stems and leaves in those plants, little is known about microbes associated with plant seeds, in particular, the true knowledge of endophytic diversity in medicinal plant seeds.

Genetic evidence suggests that uncultured microorganisms account for a high percentage of environmental microbial communities in natural ecosystems, and yet the accessible (Rappé et al., 2002), cultivable fraction is less than 1% (Epstein, 2013). Therefore, this study used metagenomics to analyze endophytic diversity in the seeds of *A*. *sinensis* and compared with endophytes isolated from healthy leaves, stems, roots and seeds of *A*. *sinensis* using 20 different media. The results (Figure 2) showed that the most frequent endophytic fungi were also *Alternaria* sp. as seen in artificial media; moreover, our results showed that endophytic fungi obtained through artificial cultivation are mainly phyla *Ascomycota*, among which none of the endophytes isolated from the *Angelica* seed belonged to *Pseudallescheria* sp. and *Fusarium* sp., as seen in the diversity analysis by high-throughput sequencing. Correspondingly, the microorganisms of *Basidiomycetes* were not isolated on artificial media. The results suggest that pathogenic fungi, including *Fusarium* sp. and *Pseudallescheria* sp., may not be inherent endophytes in plants, but are formed by transfection from the environment during plant growth. As such, the microorganisms inherent in those plants, rather than the microorganisms obtained from the outside world, may be true endophytes. In addition, the endophytic bacteria obtained by artificially separating the medium are mainly divided into two phyla, *Firmicutes* and *Proteobacteria*. Furthermore, the endophytes in *A*. *sinensis* seed contained seven phyla isolated with the high-throughput sequencing of microorganisms, and five phyla of endophytic bacteria, including *Proteobacteria*, *Actinobacteria*, *Bacteroidetes*, *Microgenomates*, and *Saccharibacteria*, were not isolated on artificial medium. Therefore, we believe the endophytes in the roots, stems and leaves of *A*. *sinensis* possibly derived from the seeds of *A*. *sinensis*, and changes in individual dominant strains of roots, stems and leaves of *A*. *sinensis* may be due to changes in growth environment of *A*. *sinensis*. Our results also suggested that seeds of *A*. *sinensis* is a good material for studying the endophytes of *A*. *sinensis*. Moreover, in another study, in order to simulate the natural environment as much as possible, we used plant extract as the medium for the microorganisms cultured. However, we found that this medium was too difficult to culture the fungus and further added potato extract as a growth factor (Beever and Bollard, 1970), which significantly increased the fungal colony rate (data not shown). This result also indicates that different separation methods are necessary if more intrinsic endophytic bacteria and endophytic fungi are to be isolated.

Reactive oxygen species (ROS), generated by triplet state oxygen reacting with other molecules, such as superoxide radicals, hydroxyl radicals, and hydrogen peroxide (H_2_O_2_), is the major sources of primary catalysts that initiate oxidation *in vivo* and *in vitro* (Du et al., 2013). When these species are produced in excess due to pathophysiological processes or environmental interference, ROS can promote tissue damage and result in disease. Antioxidants are known to prevent those reactions caused by free radicals (Goodman et al., 2011; Marson Ascencio et al., 2014). In this paper, six antioxidant activity assays, including DPPH, hydroxyl radical scavenging assay, FRAP assay, total antioxidant capacity assay, H_2_O_2_ scavenging assay and superoxide anion radical scavenging assay, were used to comprehensively evaluate the antioxidant activities of endophytic metabolites. The results showed that the sample JH-4 mycelium displayed the best antioxidant activity. Furthermore, total phenols and total flavonoids are known as the main components of antioxidant activity, and their contents are determined. Phenolic compounds are known to have very important biological activities, such as preventing hormone-related cancers, and as potent antioxidant agents (Pise et al., 2010). The antioxidant activity of phenolic compounds is mostly derived from their redox actions, neutralizing free radicals, quenching singlet and triplet oxygen, or decomposing peroxides. Flavonoids are widely distributed polyphenolic compounds and have recently received extensive attention due to their important antioxidant properties (Pietta, 2000). Flavonoids probably showed antioxidant activities because of their high total number of OH groups (Firuzi et al., 2005). According to the current results, we found a significant correlation between flavonoid content and radical scavenging effect, but the correlation between total phenolics content and antioxidant activity was relatively weaker. The results further suggest that antioxidants in the bacteria may be flavonoids. The results obtained in this study also agree with those published previously in the literature (Moein et al., 2008). In addition, there were few studies on the activity of *A*. *sinensis* endophytes besides (Yang et al., 2013). The antimicrobial activity of isolated endophytes from *A*. *sinensis* were detected using the paper disc diffusion method with five tested strains, including *E*. *coli*, *S*. *aureus*, *B*. *licheniformis*, *P*. *aeruginosa*, and *Str*. *pneumoniae*. Our study showed that YH-12-1 mycelium had a strong inhibitory effect on *P*. *aeruginosa* and *S*. *aureus*, with a MIC of 25 μg/mL, which also indicates that further research is necessary.

## Conclusion

In the present study, with 20 different media, 226 endophytes were isolated from *A*. *sinensis* and identified based on morphological characteristics and molecular biology. This study was the first of its kind to study the diversity of endophytes in the seeds of medicinal plants. Compared with high-throughput sequencing, a minority of endophytes were isolated from *A*. *sinensis*. Furthermore, results of bioactivity evaluation showed that JH-4 mycelia and YH-12-1 mycelia have good antioxidant and inhibitory activity. All of our results showed that endophytes were highly abundant in *A*. *sinensis*, and indicated that their metabolites are potential viable sources for exploring many novel active compounds. Further exploration of endophytic isolation methods to obtain as many microbial resources as possible is still necessary.

## Data Availability Statement

The datasets presented in this study can be found in online repositories. The names of the repository/repositories and accession number(s) can be found in the article/ Supplementary Material.

## Author Contributions

All authors listed have made a substantial, direct and intellectual contribution to the work, and approved it for publication.

## Conflict of Interest

The authors declare that the research was conducted in the absence of any commercial or financial relationships that could be construed as a potential conflict of interest.

## Footnotes

1 http://drive5.com/uparse/

2 http://rdp.cme.msu.edu/

3 http://www.arb-silva.de
