# Supplementary material for: Diversity and Bioactivity of Endophytes From Angelica sinensis in China
Source: Front Microbiol. 2020 Aug 18;11:1489. doi: 10.3389/fmicb.2020.01489 (PMC7461802; doi:10.3389/fmicb.2020.01489)
Supplement: Supplementary file 1 [file Data_Sheet_1.docx]

Table S1. Diversity indices of bacterial and fungal communities from 4 seed samples of *Angelica sinensis.*

|  | **Sample** | **Community Diversity index** | | |  | | **Community richness estimator** | | |
| --- | --- | --- | --- | --- | --- | --- | --- | --- | --- |
|  |  | **Ace** | **Sobs** | **Chao** |  | **Shannon** | | **Simpson** | **Coverage（%）** |
| **Fungi** |  |  |  |  |  |  | |  |  |
|  | 1 | 68.50 | 39 | 50.14 |  | 1.18 | | 0.35 | 99.98 |
|  | 2 | 85.02 | 61 | 69.75 |  | 1.46 | | 0.29 | 99.98 |
|  | 3 | 70.73 | 68 | 69.67 |  | 1.32 | | 0.34 | 99.99 |
|  | 4 | 54.44 | 52 | 55.33 |  | 1.03 | | 0.63 | 99.99 |
| **Bacteria** |  |  |  |  |  |  | |  |  |
|  | 1 | 110.44 | 81 | 104.63 |  | 0.52 | | 0.74 | 99.95 |
|  | 2 | 98.56 | 82 | 97.83 |  | 0.61 | | 0.69 | 99.96 |
|  | 3 | 108.36 | 94 | 103.00 |  | 1.23 | | 0.47 | 99.96 |
|  | 4 | 97.47 | 93 | 94.50 |  | 0.47 | | 0.82 | 100.00 |

**bacteria fungi**

**Fig. S1** Rarefaction curves of the normalized OTUs number at 97% similarity.
